# Studies on the Efficient Extraction of Ovotransferrin and the Effect of Heating Treatment on Its Structure and Activity

**DOI:** 10.3390/foods14050905

**Published:** 2025-03-06

**Authors:** Yujie Su, Qianwen Zhao, Cuihua Chang, Junhua Li, Lulu Guo, Shende Hu, Zijian Huang, Luping Gu, Yanjun Yang

**Affiliations:** 1 State Key Laboratory of Food Science and Resources, School of Food Science and Technology, Collaborative Innovation Center of Food Safety and Quality Control in Jiangsu Province, Jiangnan University, Wuxi 214122, China; suyujie@jiangnan.edu.cn (Y.S.); 6220111156@stu.jiangnan.edu.cn (Q.Z.); shangcuihua@163.com (C.C.); lijunhua@jiangnan.edu.cn (J.L.); 6230112019@stu.jiangnan.edu.cn (L.G.); 6230111184@stu.jiangnan.edu.cn (S.H.); guluping@jiangnan.edu.cn (L.G.); 2 Hunan Jiapin Jiawei Technology Development Group Co., Ltd, Hunan Engineering & Technology Research Center for Food Flavors and Flavorings, Jinshi 415400, China; 3 College of Bioscience and Biotechnology, Hunan Agricultural University, Changsha 410128, China; 13357917857@163.com

**Keywords:** Ovotransferrin, extraction, moderate heat treatment, physicochemical property, biological activity

## Abstract

Ovotransferrin (OVT) is very rich in nutritional value and possesses a variety of biological activities. However, there is a lack of suitable OVT extraction methods that are simple and suitable for large-scale production. For this reason, this study explored a new method of ovalbumin OVT extraction based on mesophilic treatment. The effects of different heat treatment conditions on the physicochemical properties and bioactivities of the prepared OVT and their influence mechanisms were investigated. The results showed that OVT could be efficiently extracted from egg white by moderate heat treatment. Based on single factor experiments, response surface methodology was used to determine the effects of heat treatment time, temperature and pH on the extraction rate of OVT. The yield was 93.65 ± 0.53% under the optimal extraction conditions (62.5 °C, 75 min, pH 8). SDS-PAGE and FT-IR showed that changes in the influencing factors during heating had different effects on OVT. In addition, different extraction parameters had different effects on the iron-binding and antioxidant capacities of OVT. This study provides a fast and efficient preparation method for OVT from egg white, which lays the foundation for the wide application of OVT.

## 1. Introduction

Ovotransferrin (OVT), also known as conalbumin, is a soluble, non-crystalline glycoprotein derived from egg white. OVT consists of 686 amino acid residues and accounts for 12–13% of the total protein content of egg white. It has an isoelectric point of about 6.0 and a molecular weight between 70 and 78 kDa [1]. Current research suggests that OVT shows great potential for application in food preservation, the delivery of nutrients and as a functional food ingredient [2,3,4,5]. As the major iron-binding protein in egg white proteins, OVT shows remarkable structural similarity to lactoferrin (LTF). In particular, both have comparable iron-binding sites, similar amino acid sequence and overall structural folding of the molecule. Therefore, OVT carries out many biological functions similar to those of LTF, such as antibacterial, antioxidant, antiviral, immunomodulatory and iron absorption-promoting activities [3,6,7]. Moreover, OVT is a high-abundance protein in egg white which is available to be extracted in large quantities and at low cost.

Most of the reports of isolations and purifications of OVT from egg whites were limited to small laboratory scale, including precipitation using ammonium sulfate and ethanol, chromatography and resin methods and membrane separation [8,9]. These traditional protein isolation and purification methods usually have many problems. For example, the use of organic reagents can induce varying degrees of denaturation in OVT, leading to a notable decline in its biological activity. Moreover, the purity of OVT extracted through ultrafiltration and nanofiltration is often suboptimal. Additionally, chromatographic separation imposes stringent demands on both the properties of the packing material and the operational conditions, resulting in elevated production costs and limited processing capacity. As a result, most of the existing separation and purification methods of OVT are only suitable for laboratory scale, and it is difficult to realize large-scale industrialization. The high production costs, time-consuming nature, labor-intensiveness and low purity and activity of the product have greatly hindered the industrial production of OVT. Therefore, the study of a simple, rapid and efficient new method for the preparation of OVT which has the potential to be used in industrial scale has become a research hotspot at present.

Heat treatment is currently one of the most common processing methods for denaturing proteins. Heat treatment can induce changes in protein structure, including the folding of natural secondary and tertiary structures, exposure of sulfhydryl and hydrophobic groups and aggregation between proteins. Heated protein molecules may cause protein aggregation under the influence of electrostatic and hydrophobic interactions and disulfide bond bridging [10]. Recent studies have examined structural and functional changes in egg white proteins under heat treatment processing conditions. Iesel Van der Plancken et al. developed kinetic models for physicochemical changes during thermal treatment, predicting industrial outcomes like solubility variations and gelation behavior [11]. Minmin Ai et al. showed that the ultrasound–thermal combination enhances emulsification in hydrolyzed egg white peptides. Analyses revealed improved oil-in-water emulsion stability through better dispersibility and reduced viscosity [12]. Additionally, their research demonstrated that calcium hydroxide (Ca(OH)_2_) addition during heating strengthens NaOH-induced duck egg white gels via enhanced cross-linking mechanisms [13]. Compared to other extraction methods, heat treatment is simple, low cost and does not require the use of organic reagents. After removing ovomucin (OVM) and lysozyme (Lys) during egg white pretreatment, the difference in thermal denaturation temperatures of ovalbumin (OVA) and OVT in egg white was utilized to achieve separation by heating at a specific temperature, time and pH to cause aggregation and precipitation of OVT, thus obtaining relatively high yields of OVT and realizing efficient extraction of OVT.

In this experiment, the method of moderate heat treatment was adopted for the preparation of OVT. The process parameters for the preparation were investigated according to the yield and purity of OVT. The effect of heating conditions on the physicochemical properties and biological activity of OVT were also investigated. This study provides a theoretical basis for optimizing the extraction process of OVT and lays the foundation for developing OVT as functional materials in the future.

## 2. Materials and Methods

### 2.1. Materials

The commercially available hen eggs were supplied by the local supermarket (Wuxi, China). Weakly acidic cationic ion exchange resins was obtained from Kangde Egg Industry (Nantong, China). Electrophoresis protein marker and 4× protein sampling buffer were purchased from Beijing Soleberg Technology Co., Ltd. (Beijing, China). All reagents used in the study were of analytical grade unless otherwise stated.

### 2.2. Preparation of H-Ovotransferrin

The egg white was separated from fresh eggs, and the colloidal structure was disrupted by slowly stirring for 40 min at 4 °C. The pH of the egg white was adjusted to 6.0 with 1 M citric acid. After stirring in a magnetic mixer at 4 °C for 3.5 h, the egg white was centrifugated at 8000× *g* for 15 min and the supernatant was stored in a 4 °C refrigerator for standby. The pH of the supernatant was adjusted to 8.0 by 1 M NaOH and centrifuged again at 10,000× *g*, 4 °C for 20 min. After removing the insoluble material, cation-exchange resin was added to absorb the lysozyme in egg white, the amount of resin added was selected to be 1:4 (resin/egg white, *w*/*w*) and the adsorption time was 90 min. The adsorbed supernatant was collected and stored in a refrigerator at 4 °C. The adsorbed supernatant was adjusted to different pH (5, 6, 7, 8, 9), then placed in a constant temperature water bath with different temperature (60 °C, 62.5 °C, 65 °C, 67.5 °C, 70 °C) and heated for different times (10 min, 30 min, 60 min,90 min, 120 min, 180 min), respectively. Then, the samples were cooled immediately and centrifuged at 8000× *g*, 4 °C for 20 min. The precipitate (named as H-ovotransferrin, H-OVT) was collected, then rinsed with deionized water for three times and freeze-dried to obtain the H-OVT dry powder.

### 2.3. Extraction Yield of H-OVT

The proteins in the original egg white and lyophilized sample were determined according to the Kjeldahl method (GB5511-1985) by the conversion factor (N% × 6.25) [14]. The OVT extraction rate (EY%) was calculated according to the following formula:

(1) EY%=OVT in lyophilized sample/total content of OVT in egg white×100%

### 2.4. Single Factor Experiments

For the heat treatment extraction of OVT, there are three main influencing factors such as heating time, heating temperature and pH value. In this study, heating time (30 min, 60 min, 90 min, 120 min, 180 min), heating temperature (60 °C, 62.5 °C, 65 °C, 67.5 °C, 70 °C) and pH (5, 6, 7, 8, 9) were used as the three factors. The OVT yield was used as an indicator, and single factor experiments were performed sequentially according to Section 2.2 and Section 2.3. The optimal condition obtained from each experiment was one of the conditions for the next single factor test.

### 2.5. Response Surface Experimental Design

Based on the results and the single factor experiments, temperature (A), time (B) and pH (C) were used as the three experimental factors with OVT yield as the response value. The optimal range of each factor was chosen to design the three-factor and three-level response surface experiments (Table 1). Regression fitting of the factors was performed using Design-Expert 12 (v12.0.3.0) software to optimize the OVT extraction conditions.

### 2.6. Determination of Solubility

The solubility of H-OVT was determined according to the biuret method. An amount of 0.5 g sample was dissolved in 10 mL of deionized water, and swirled until the sample was dissolved completely. After that, the sample solution was centrifuged at 7000× *g* for 15 min. The supernatant was mixed with the biuret reagent for half an hour. The absorbance of the solution was measured at 540 nm with a UV-visible spectrophotometer (UH-5300, Hitachi Corporation, Tokyo, Japan). The protein concentration of the sample was determined using the equation of the standard curve. The standard curve of bovine serum albumin (BSA) was used in this experiment.

### 2.7. SDS-PAGE

The protein composition of the sample was analyzed by sodium dodecyl sulfate polyacrylamide gel electrophoresis (SDS-PAGE). The electrophoresis was operated at a constant voltage of 80 V using 10% separating gel and 5% stacking gel. After that, the gel was colored for 20 min using Coomassie Brilliant Blue R-250, and then decolorized until the bands were clear. The purity of the OVT was calculated by converting the density of protein bands in the gel picture using the ImageJ 1.50 (NIH, Bethesda, MD, USA) as the percent of the total gel density [15].

### 2.8. Particle Size, Zeta Potential and PDI Measurements

The lyophilized H-OVT samples were dissolved in ultrapure water to prepare a protein solution at a concentration of 1 mg/mL with a testing temperature of 25 °C. According to the method of Zhang et al. [16]. The particle size and particle dispersion of H-OVT was determined with a Malvern Mastersizer 2000S (Malvern Instruments Ltd., Malvern, UK). And the Zeta potential of H-OVT was determined using a Nano-ZS nanosize analyzer (Zetasizer nano ZS, Malvern, UK). The refractive index of the particles was 1.763, and the refractive index of the phosphate buffer was 1.33.

### 2.9. Fourier-Transform Infrared Spectroscopy

The secondary structure of H-OVT protein was measured and analyzed using a Fourier-transform infrared spectrometer (FTIR IS10, Nicolet, Madison, WI, USA) according to the method of Tang et al. [17]. The H-OVT sample powder was taken and mixed and ground with spectroscopic grade KBr at a mass ratio of 1:75. Each sample was run three parallel experiments with a full waveband scan (4000 to 400 cm^−1^) with 32 scans at a resolution of 4 cm^−1^. Basic infrared spectrum analysis, peak marking and spectrum collecting were performed with Omnic 8.2 software. Second-order derivatives were carried out in the spectral band range using Peakfit Version 4.12 software, and the relative percentages of each secondary structure component were calculated [18].

### 2.10. Biological Activity of H-OVT

#### 2.10.1. Determination of Iron-Binding Capacity 

The iron-binding capacity of H-OVT was measured according to the method of Decker, with a slight modification [19]. An amount of 1 mL of H-OVT sample solution (1 mg/mL) was added to 3 mL of deionized water. Then, 0.1 mL of 1 mM FeSO4 and 0.2 mL of 5 mM phenanthrozine were added. The reaction was carried out at 25 °C for 20 min. Then, the absorbance of the solution was measured at 465 nm. The iron-binding rate was calculated according to the following equation.

(2) $$Iron-binding\;rate\;\%\;=\left(1-A_{1}/A_{0}\right)\times 100\%$$

where A_1_ is the absorbance of the H-OVT sample, A_0_ is the absorbance of the sample replaced with deionized water.

#### 2.10.2. Determination of Antioxidant Capacity

##### Hydroxyl Radical Scavenging Ability

Hydroxyl radical scavenging capacity of H-OVT was carried out according to Ma et al., with a slight modification [20]. The H-OVT sample was dissolved in deionized water to prepare a solution of 1 mg/mL. Then, 1 mL of 9 mM ferrous sulfate, 1 mL of 9 mM salicylic acid–ethanol solution and 1 mL of 9 mM hydrogen peroxide were added into the solution to initiate the reaction. After mixing well, the samples were incubated in a 37 °C water bath for 30 min and then measured the absorbance at 510 nm. The same concentration of vitamin C was used as the control group. Hydroxyl radical scavenging rate was calculated using the following equation.

(3) $$Hydroxyl\;radical\;scavenging\;rate\%=\left[1-(A_{1}-A_{2})/A_{0}\right]\times 100\%$$

where A_0_ is the absorbance of the control (without sample), A_1_ is the absorbance of the samples and A_2_ is the absorbance without sodium salicylate.

##### DPPH• Scavenging Activity

DPPH• scavenging activity was measured by the method of Ma et al., with some modifications [20]. The H-OVT sample was dissolved in deionized water to prepare a solution of 1 mg/mL. Then, 4 mL of 0.2 mM DPPH was added into 1 mL sample solution and mixed well. After reacting in the dark for 30 min, the sample was centrifugated at 8000× *g* for 10 min to remove the precipitate. Additionally, 95% ethanol was used as a reference to measure the absorbance at 517 nm. The same concentration of vitamin C was used as the control group. DPPH• scavenging activity was calculated using the following equation.

(4) $$DPPH\cdot scavenging\;rate\;\%\;=(A_{0}-A_{1})/A_{0}\times 100\%$$

where A_0_ is the absorbance of control groups and A_1_ is the absorbance of the sample.

##### ABTS^+^ Scavenging Activity

The ABTS^+^ free radical scavenging capacity of H-OVT was determined according to the method of Ma et al., with slight modifications [20]. The ABTS^+^ stock solution was obtained by mixing 7 mM of ABTS^+^ solution with 2.45 mM of potassium persulfate solution in equal volume and left to stand overnight at 25 °C protected from light. Then, the ABTS^+^ working solution was obtained by diluting the ABTS^+^ stock solution with methanol so that the absorbance at 734 nm was 0.70 ± 0.02. An amount of 100 μL of the H-OVT sample (1 mg/mL) was mixed with 2 mL of ABTS^+^ working solution and incubated at 25 °C for 30 min, then the absorbance was measured at 734 nm. The same concentration of vitamin C was used as the control group. ABTS^+^ scavenging capacity was calculated using the following equation.

(5) $${ABTS}^{+}\;scavenging\;rate\;\%\;=(1-A_{1}/A_{0})\times 100\%$$

where A_0_ is the absorbance of the ABTS^+^ working solution; A_1_ is the absorbance after sample addition.

### 2.11. Statistical Analysis

Data were analyzed using statistical software (SPSS version 22, SPSS, Chicago, IL, USA) and expressed as mean value ± standard deviation. The statistical significance among sample groups was calculated using one-way ANOVA and Tukey’s test. Three determinations of each sample were executed, with the significance level set at *p* < 0.05.

## 3. Results

### 3.1. Effect of Three Factors on Purity of H-OVT

**Figure 1:**
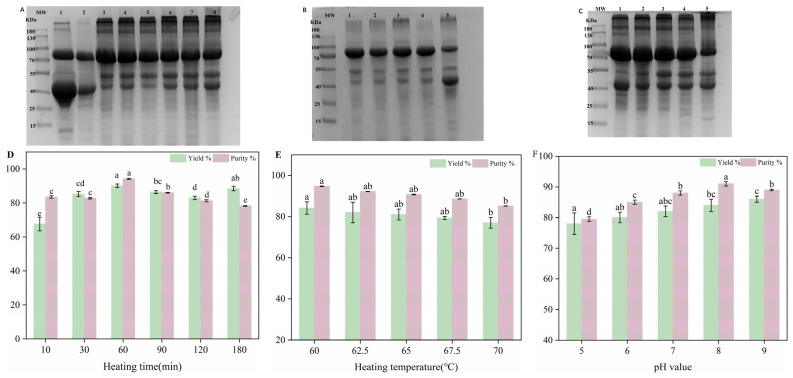
Electropherograms of original egg white (band 1), pretreated egg white (band 2), OVT obtained at 10 min, 30 min, 60 min, 90 min, 120 min, 180 min (band from 3 to 8) (**A**), electropherograms of OVT obtained at 60 °C, 62.5 °C, 65 °C, 67.5 °C, 70 °C (band from 1 to 5) (**B**), electropherograms of OVT obtained at pH5, pH6, pH7, pH8, pH9 (band from 1 to 5) (**C**), protein yield and purity of H-OVT prepared at different heating time (**D**), temperature (**E**) and pH (**F**). Different lowercase letters above bars indicate significant differences (Tukey’s HSD test, *p* < 0.05).

SDS-PAGE was used to verify the composition of protein samples and calculate their purity. As shown in Figure 1A band 1, four main protein bands were in egg white, corresponding to OVT, OVA, OVM and Lys, respectively. The results showed that the H-OVT was obtained when the egg white was heated at 60 °C and pH 8 for different times after two steps of pretreatment. During the egg white pretreatment process, OVM (pI = 6) was removed by isoelectric precipitation. The disappearance of the band corresponding to Lys from bands 1 and 2 indicated that the pretreatment could effectively eliminate Lys. At a pH of 8.0, Lys carried a positive charge as its isoelectric point was greater than 8.0, while other proteins in egg white carried negative charges. Therefore, Lys in egg white could be adsorbed effectively by cation exchange resin at this pH condition. After two steps of pretreatment, the remaining proteins in the egg white were mainly OVT and ovalbumin (OVA). As the denaturation temperature of OVA (about 72 °C) was much higher than OVT (about 63 °C), the OVT was much easier to accumulate during moderate heat treatment. Therefore, the combination of moderate heat treatment and two steps of pretreatment could be used to extract H-OVT with a higher purity [21].

Figure 1A showed the electropherograms of H-OVT produced by different heat times. OVT had poor heat resistance, with a denaturation temperature of 60 °C, while OVA had better heat resistance, with a denaturation temperature of 84 °C. Because of the difference in heat resistance between OVT and OVA, they can be separated effectively through heat treatment with moderate temperature. In addition, a small number of macromolecular polymers with a molecular weight larger than 170 KD appeared at the top of the gel. The reason for this phenomenon may be that OVT formed polymeric substances at higher temperatures. Moreover, OVT and OVA may increase in relative molecular weight because of cross-linking and aggregation at high temperatures, which prevented the aggregates from passing through the concentrated gel. When the sample was heated for 10 min, more than one protein band was present in H-OVT products. It has been reported that the protein fractions in egg white were chimerized through a noncovalent hydrophobic interaction [22]. The other proteins may not yet dissociate and precipitated together with OVT during the short heating time. Meanwhile, new bands appeared between 40 and 55 kDa after heating, and it was speculated that this might be due to the co-action of OVA and OVT to generate new aggregates after heat treatment. With the extension of the heating time, the bands of impurity proteins gradually decreased, and eventually, the main band of impurity protein corresponds to ovalbumin, with its content continuously increasing. This may be due to the gradual decrease in the solubility of OVA with the increasing heating time. When OVA was in a monomeric state, its solubility decreased slightly after heating. A more significant decrease in solubility may have occurred when it was present with OVT, and potential interactions may occur after heating. This result was similar to the previous studies, which showed that the solubility of OVA and ovomucin (OVM) decreased slightly when heated separately at 60 °C and 65 °C, and decreased significantly at 70 °C. While the solubility of OVA and OVM after co-heating decreased continuously with the extension of heating time, which may also be related to their interaction [23].

Figure 1B showed the bands of H-OVT samples obtained by heating in the range of 60 °C to 70 °C. The results indicated that H-OVT with high purity can be obtained at the temperature range from 60 °C to 70 °C. When the heating temperature was less than 60 °C, OVT did not undergo thermal aggregation, and when the heating temperature was greater than 70 °C, OVT was in the gel state. With the increase in heating temperature, the content of OVA and ovoglobulin contained in the precipitate increased, which was because the heating temperature reached the thermal solidification point of both proteins, the aggregation occurred and the solubility gradually decreased.

Figure 1C shows the bands of H-OVT samples obtained by regulating the pH of egg white in the range of pH 5 to pH 9. It can be clearly observed that the purity of H-OVT was the highest when the egg white was neutral. When the egg white was under acidic or alkaline conditions, the content of heteroprotein was higher, which may be due to the difference in the isoelectric point of OVT and OVA. When the solution was acidic, it was close to the isoelectric point of OVA (PI = 4.8), which made OVA easy to precipitate. On the contrary, in an alkaline environment, it was equally easy to cause protein denaturation and precipitation.

### 3.2. Effect of Three Factors on Yield of H-OVT

The protein yield of H-OVT extracted at different heating times were shown in Figure 1D. The protein yield of the OVT obtained increased as the heating time extended. This was due to the thermal aggregation of OVT that occurred when it was heated at certain temperature. The longer the heating time, the higher the degree of denaturation of OVT, and then the more flocculation precipitation due to the denaturation of protein, so that the protein yield in the obtained H-OVT was increasing. When the egg white was heated for 60 min, the protein yield of the H-OVT reached the maximum value, and as the heating time continued to be extended, the total protein yield did not change significantly, which indicated that the OVT in the egg white was almost completely denatured and was extracted thoroughly. Therefore, 60 min was selected as the optimum heat treatment time.

The protein yield of H-OVT extracted at different heating temperatures is shown in Figure 1E. With the increase in heating temperature, the protein yield of the obtained OVT gradually decreased (*p* < 0.05), which was due to the fact that when heated at 60 °C for 60 min, the OVT in the egg white was almost completely denatured and transformed into precipitate. As the heat treatment temperature continued to increase, the protein yield of H-OVT gradually decreased, which was due to the fact that the complete precipitation of OVT was basically achieved when heating at 60 °C for 60 min, and further increasing the temperature had little effect on the precipitation of OVT, but it would result in an increase in OVA precipitation, which was unfavorable to OVT extraction. Therefore, 60 °C was selected as the optimum heat treatment temperature.

The protein yield of H-OVT extracted by heating under different pH conditions is shown in Figure 1F. The protein yield of the obtained H-OVT increased with an increase in the pH of the egg white (*p* < 0.05), which is due to the fact that excess alkaline could lead to protein denaturation and precipitation. Therefore, pH 8 was selected as the optimal pH for heat treatment.

### 3.3. Response Surface Optimization of OVT Conditions for Heat Treatment Extraction

Orthogonal test results for extraction of OVT by heat treatment (Table 2) and multiple regression analysis were performed using Design-Expert 12 software and the following regression equation was obtained:

$$Yieid\%=93.97+0.53\times A-0.25\times B+0.58\times C+0.36\times A\times B-0.14\times A\times C+0.34\times B\times C-7.01\times A^{2}-1.75\times B^{2}-3.55\times C^{2}$$

where A = time, B = temperature, C = pH.

### 3.4. Model Analysis of Variance Results

The results of the model ANOVA are shown in Table 3. The F value was used to reflect whether the model is significant or not. The larger the value, the more significant the model is. In this experiment, the F value was 80.64, which indicated that the model was significant. The *p*-value was used to reflect the significance of the effects of various factors in the model. Statistical significance was defined using standard alpha thresholds: terms with *p* < 0.05 were considered statistically significant, and those with *p* < 0.001 provided stronger evidence against the null hypothesis (α = 0.001). According to Table 3, the factors affecting the yield of OVT were pH > time > temperature. r2 = 0.9962, R2adjusted = 0.9895 and the lack of fitting term was not significant (*p*-value > 0.05), which suggests that the model was well fitted to analyze and predict the conditions of heat treatment for extracting OVT. It also demonstrated that the experimental methodology was reliable.

### 3.5. Confirmation and Verification of the Optimal Conditions of Response Surface

**Figure 2:**
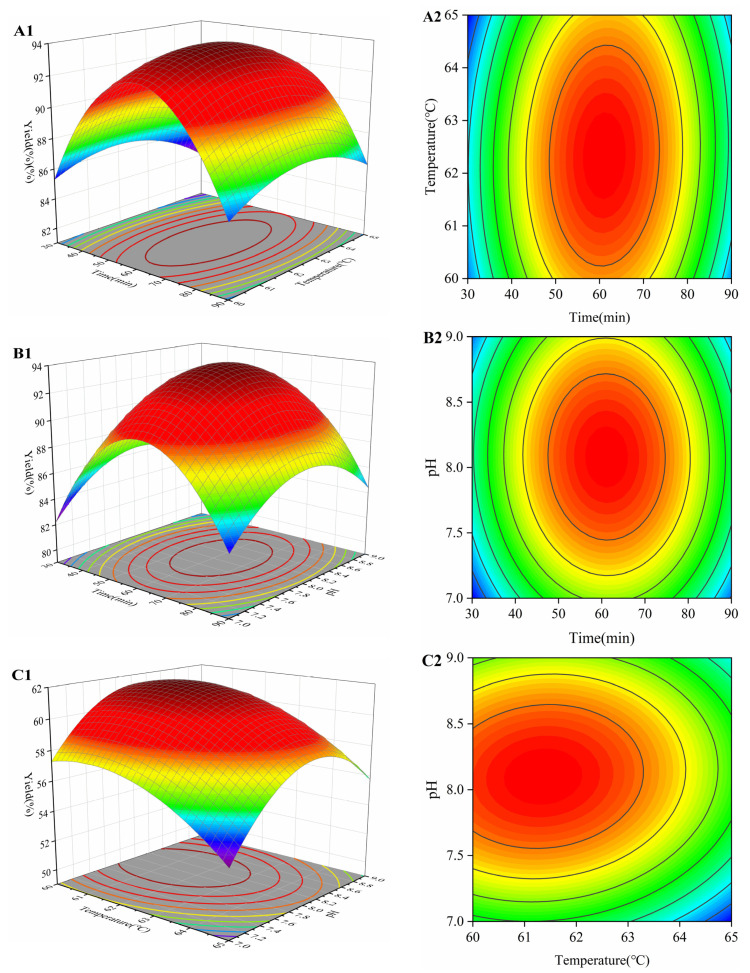
3D response surface plots (**A1**,**B1**,**C1**) and contour plots (**A2**,**B2**,**C2**) of the effects of time, temperature, and pH on the yield. (**A1**,**A2**) indicate the effects of time and ask, (**B1**,**B2**) indicate the effects of time and pH, and (**C1**,**C2**) indicate the effects of temperature and pH.

Three-dimensional plots of any two-factor interaction can be obtained from this model. As shown in Figure 2, the response surface plot was steep, indicating that there was some interaction between time, temperature and pH, respectively. The optimal extraction process was analyzed by Design-Expert 12.0, and the temperature was determined to be 62.48 °C, time 65.51 min and pH 8.09, at which the highest theoretical yield could be achieved. Considering the operability of the experimental setup, the enzyme digestion process was modified: the enzyme digestion temperature was 62.5 °C, time 65 min and pH 8. In order to verify the scientific validity of the response surface model, three repetitions of the experiment were carried out, and the average yield was 93.65 ± 0.53%, which was close to the predicted value.

### 3.6. Protein Solubility of H-OVT

**Figure 3:**
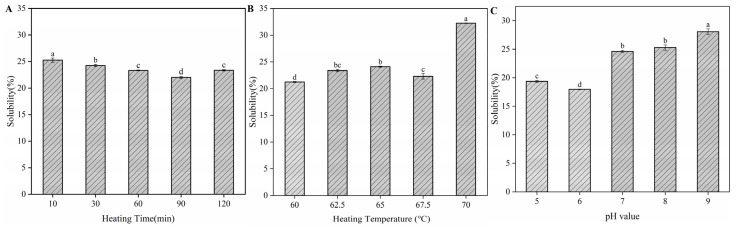
Protein solubility of H-OVT prepared at different heating times (**A**), temperatures (**B**) and pH (**C**). (Different lowercase letters indicate statistically significant differences between groups).

Figure 3 illustrates the solubility of H-OVT extracted through different heating times, temperatures and pH. It was known that the solubility of natural OVT (N-OVT) was more than 90%. As shown in Figure 3, the solubility of H-OVT was generally low, which was mainly due to the thermal denaturation of OVT, resulting in changes in its structure. Most water-soluble globular proteins unfold their structure upon heating, exposing hydrophobic groups within the protein, and the protein aggregates through hydrophobic interactions, leading to the decreasing of solubility. The OVT structure unfolded, and the buried hydrophobic amino acids were exposed as the heating time increased. The hydrophobic interactions promoted the aggregation of OVT molecules, which decreased their solubility. However, the solubility of OVT increased when the egg white was heated for 120 min (*p* < 0.05) (Figure 3A). This may be because a large number of charged amino acids were transferred to the surface of the monomer/polymerized protein molecules when OVT was heated for a relatively long time (120 min). These charged amino acids enhanced the electrostatic repulsion between the protein particles, thus inhibiting the aggregation of the protein particle molecules and increasing the solubility [24]. This was similar to the results reported by Kitabatake et al., who found that the increase in electrostatic repulsion can inhibit aggregation between protein molecules [25].

From Figure 3B, it can be found that the solubility of H-OVT increased slowly with the increase in heating temperature in the range of 60 °C to 65 °C (*p* < 0.05). This may be due to the gradual unfolding of the protein structure during moderate heating, which caused the loosening of the OVT structure but did not expose the hydrophobic groups buried inside the protein at these conditions. The decrease in the solubility of H-OVT when the egg white was heated at 67.5 °C may be related to the fact that the heating temperature reached a critical point. When at this critical point, the hydrophobic groups within the protein molecules were exposed, which was consistent with previous findings [26]. A significant increase in the solubility of H-OVT was observed when the temperature was higher than 67.5 °C (*p* < 0.05), which may be attributed to the fact that during the rapid cooling process after heating, the larger temperature difference led to the rapid contraction of the protein structure, and the soluble or insoluble aggregation of the protein occurred. During the aggregation process, some of the hydrophobic groups were once again encapsulated inside the molecule, which led to a decrease in hydrophobicity, and hence an increase in solubility [24].

As shown in Figure 3C, the lowest solubility of H-OVT was observed when the pH was 6, which suggested that heating may shift the isoelectric point of OVT slightly. The solubility of H-OVT decreased with increasing pH when the solution pH was less than the isoelectric point and increased with increasing pH when the solution pH was greater than the isoelectric point (*p* < 0.05). Prior studies demonstrate pH-dependent heat-induced aggregation of antineutral to weakly acidic conditions (pH 5–7): near its isoelectric point (pI), charge neutralization enhances hydrophobic interactions, promoting aggregation. Alkaline conditions (pH 9): further from pI, increased surface negative charges induce electrostatic repulsion to inhibit aggregation [27]. On the other hand, when the pH is away from the isoelectric point, proteins contain net negative or positive charges and provide more water-binding sites, which leads to the increase in protein solubility. Typically, the lowest protein solubility was found when the pH was near to the isoelectric point, which resulted in the reduction in the electrostatic repulsion between molecules and minimal interaction with water. Thus, water–protein interactions were replaced by protein–protein interactions.

### 3.7. Particle Size, Zeta Potential and PDI of H-OVT

**Figure 4:**
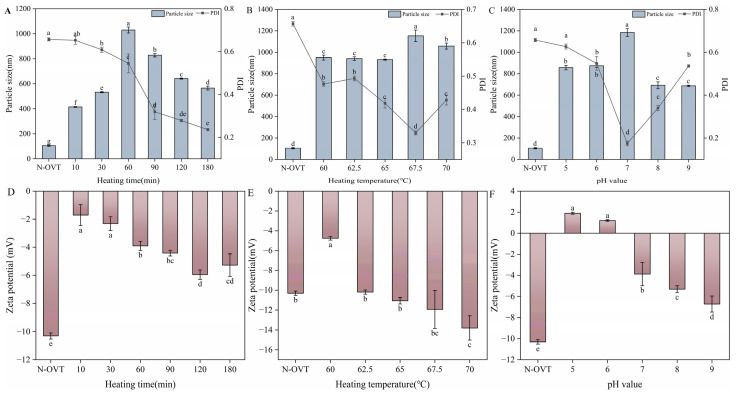
The particle sizes and PDI of the fabricated H-OVT at different heating times (**A**), temperature (**B**) and pH (**C**), Zeta potential of the fabricated H-OVT produced by different heating times (**D**), temperature (**E**) and pH (**F**). (Different lowercase letters indicate statistically significant differences between groups).

Figure 4 showed the particle size and PDI of H-OVT. Overall, the particle size of H-OVT was about 300–1000 nm, which was significantly higher than that of N-OVT (100–120 nm). It can be inferred that a part of the molecular structure of the OVT underwent irreversible changes after thermal aggregation, which resulted in an increase in the particle size.

The protein particle size increased as the heating time increased at the first 60 min (*p* < 0.05) (Figure 4A). This may be due to the aggregation of protein molecules as a result of heating. After that, the protein particle size decreased significantly with the extension of heating time (*p* < 0.05). This may be due to the fact that as the protein structure unfolded, the charged amino acids were exposed to the protein surface, which enhanced the electrostatic repulsion between the proteins. Although hydrophobic interaction could promote protein aggregation, electrostatic repulsive force inhibited protein aggregation. When the electrostatic repulsive force was greater than the hydrophobic force, the particle size of the protein began to decrease.

As shown in Figure 4B, when the egg white was heated at 67.5 °C, the H-OVT showed the largest particle size. This was consistent with the result of solubility shown above. However, the particle size of H-OVT decreased when the heating temperature was higher than 67.5 °C. This may be because of the thermal depolymerization of large-sized aggregates caused by high-temperature heat treatment.

The isoelectric point of OVT was 6.5, where the aggregates would be produced, resulting in the largest protein particle size at the isoelectric point. However, as shown in Figure 4C, the average particle size of OVT was largest at pH 7. This may be related to the unfolding of its three-dimensional structure. Due to the charge balance of H-OVT under the condition that the pH of the solution was equal to the isoelectric point, the intermolecular interaction forces were weak, resulting in a looser structure, which exhibits a larger particle size. Under acidic conditions, the amino terminus and lysine residues of the H-OVT may be positively charged, while the carboxy terminus and aspartic acid residues were negatively charged, allowing for a more compact structure. Conversely, under alkaline conditions, the amino terminus and lysine residues of proteins were negatively charged, while the carboxyl terminus and aspartic acid residues were positively charged, again leading to a compact protein structure [28].

The polydispersity index (PDI) quantifies the uniformity of particle size distribution. A smaller PDI indicates that the particle size distribution is narrower, and the solution is more homogeneous and stable. As shown in Figure 4A, when the heating time was between 10 and 60 min, PDI > 0.4, indicating extensive polydispersity. When the heating time was longer than 60 min, PDI < 0.4 indicated moderate polydispersity. This result was consistent with the findings reported by Sponton et al. [29]. Furthermore. Figure 4B exhibited a gradual decrease in PDI with an increase in heating time, indicating that the protein solution system was more stable by heating. This conclusion was consistent with the previous study [29]. However, when the temperature was 70 °C, the PDI was significantly elevated again. This phenomenon may be attributed to the fact that PDI was related to protein solubility. Larger values of PDI indicated better dispersion and higher solubility of protein in water, corresponding to the section above describing solubility. The PDI was greater in acidic and alkaline conditions compared to neutral conditions, which explained the instability of OVT in acidic and alkaline conditions.

Zeta potential is a characterization of the electric potential in colloidal systems, and has a strong influence on the stability of foams and emulsions produced from protein solutions. Figure 4D–F showed the zeta potential of H-OVT produced at different heating temperatures, time and pH. It can be observed that the absolute value of zeta potential tended to increase significantly in general (*p* < 0.05). The increase in surface charge of proteins may be due to the unfolding of egg white protein structure caused by heating. This induced the outward transfer of buried charged amino acids, thus resulting in an increase in the surface charge. This result was the same as the previous study [30]. The more the surface charges, the more the protein aggregation is inhibited, making the system more stable. In addition, the potential is positive when the pH values were 5 and 6.

When the pH was greater than 6, the potential was negative. Since the isoelectric point pH of OVT was 6.5, the zeta potential was theoretically 0 at this pH.

### 3.8. Fourier-Transform Infrared Spectroscopy of H-OVT

**Figure 5:**
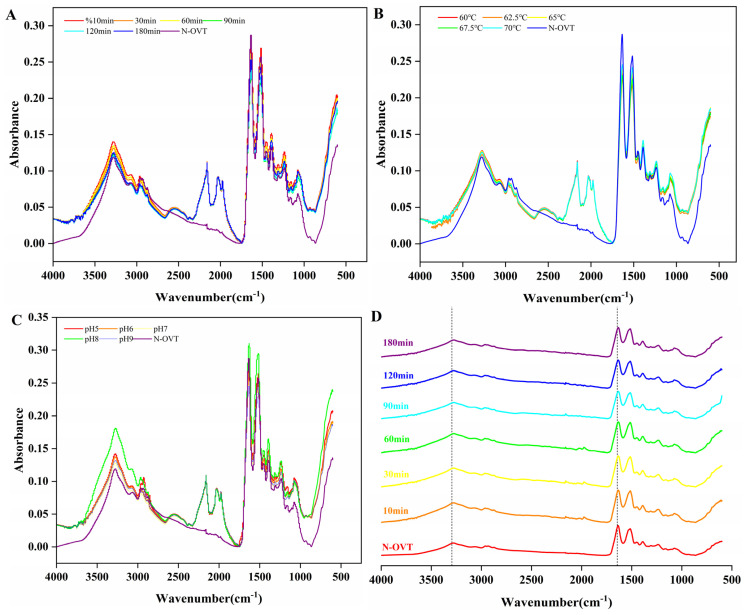

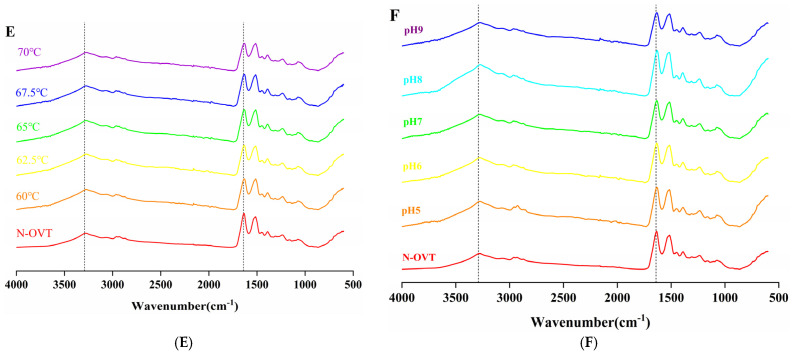
FTIR spectra of OVT obtained by heat treatment at different times (**A**,**D**), temperature (**B**,**E**) and pH (**C**,**F**), Secondary structure occupancies of OVT obtained by heat treatment at different time (**G**), temperature (**H**) and pH (**I**).

FT-IR spectroscopy was utilized to investigate the effects of heating time, temperature and pH on the secondary structure of the resulting H-OVT. As shown in Figure 5, overall, the FT-IR spectra of the OVT obtained from different conditions of heat treatment did not show significant differences compared to the natural OVT (N-OVT). The intensity of the absorption peaks of H-OVT were enhanced to different degrees at around 3300 cm^−1^ (Figure 5A–C). As shown in Figure 4A–C, H-OVT exhibited new peaks in the 2500–1700 cm^−1^ region compared to N-OVT. These spectral changes may result from heat-induced secondary structural rearrangements (e.g., increased β-sheet content) or enhanced carboxylic acid vibrations due to hydrophobic group exposure. Heat treatment induces structural changes in OVT, such as the unfolding of α-helices or the formation of β-sheets, leading to alterations in the peak shape or position of the amide I band (1600–1700 cm^−1^). Previous studies have demonstrated that thermal treatment of ferritin significantly reorganizes its secondary structure, increasing β-sheet content while reducing α-helical content, which may explain the observed shifts or splitting of the amide I band [31]. Additionally, free sulfhydryl groups (–SH) typically exhibit characteristic peaks at 2550–2600 cm^−1^ (S–H stretching vibrations). However, heat treatment likely promotes the oxidation of –SH groups to disulfide bonds (S–S), leading to a reduction in free –SH signals and the emergence of new peaks associated with oxidation products (e.g., S–O vibrations) [32]. Furthermore, thermal exposure may disrupt the hydrophobic core of OVT, enhancing the vibrations of carboxylic acid groups (–COOH) or side-chain amino groups (–NH), thereby generating new peaks in the 1700–1750 cm^−1^ or 2500–2600 cm^−1^ regions [33]. In contrast, N-OVT retains its native conformational integrity, adopting a tightly folded globular structure. Hydrophobic groups remain buried within the core, resulting in weak or masked vibrational signals from polar groups (e.g., carboxylic acids or amino groups). The peaks here were attributed to O-H stretching vibration, N-H stretching vibration and intramolecular and intermolecular hydrogen bonding formed by the O-H group in the binding water and C=O in the amino acid. This result indicated that a large number of intramolecular or intermolecular hydrogen bonds may exist in the H-OVT molecular polymers [34]. The secondary structure vibrational bands were mainly in the amide I region (1700–1600 cm^−1^), including α-helix, β-sheet, β-turning and random coil. Therefore, the amide I region is commonly used to analyze protein secondary structure. From the peak position change analysis, the amide I band of N-OVT was around 1636 cm^−1^. The amide I band was shifted to lower wave numbers after heating treatment (Figure 5D–F). This may be due to the formation of intermolecular hydrogen bonds between free amino acid residues as the globular folded structure of OVT unfolds during thermal denaturation. The electron cloud density of C=O decreased when the hydrogen bonding was strong, resulting in a shift in the absorption peak position in the low wave number direction. There were significant differences in the changes in the various parts of the secondary structure of the OVT produced under different heating conditions (Figure 5G–I). The α-helix was the most stable structure in the secondary structure of the protein, and the stability of the protein structure decreased with the increase in heating time. Therefore, thermal denaturation of OVT led to the gradual breaking of hydrogen bonds in the α-helix. Compared with N-OVT, the α-helix content of H-OVT showed a decreasing trend. Among them, the prolongation of the heating time had a smaller effect on the α-helix content, which only decreased by 0.41% (Figure 5G). Whereas, after heating at different temperatures, the α-helix content appeared to significantly decrease, by 13.64% (Figure 5H). With the increase in pH, the α-helix content decreased significantly from 16.71% to 14.91% (Figure 5I). The content of β-sheet was substantially increased compared to N-OVT. This phenomenon may be attributed to the weaker electrostatic interactions and stronger hydrophobic interactions generated in the OVT molecules during the heating phase, which enhanced the folding rate of the protein structure. Whereas heat treatment accelerated the conformational transition of the protein, leading to the faster formation of antiparallel β-sheet [35]. In addition, it has been reported that β-sheet is usually associated with the aggregation state of the protein. A larger percentage of β-sheet occupancy indicated a higher degree of protein aggregation [36]. On the other hand, the β-turn content decreased and the random coil remained essentially unchanged. These results suggest that heating induced a combination of hydrophobic interactions, intramolecular hydrogen bonding and van der Waals forces to misfold the OVT.

### 3.9. Iron-Binding Capacity of H-OVT

**Figure 6:**
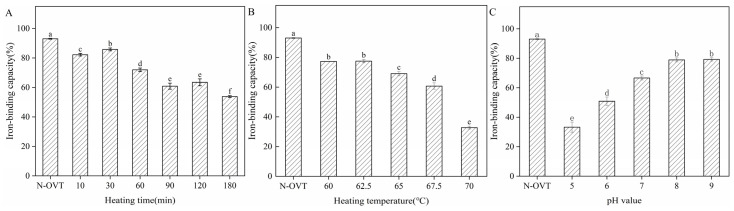
Iron-binding capacity of OVT obtained by heat treatment at different times (**A**), temperature (**B**) and pH (**C**). (Different lowercase letters indicate statistically significant differences between groups).

The iron-binding capacity of H-OVT produced by moderate heating is shown in Figure 6. As shown in Figure 6A,B, the iron-binding rate declined with an increase in heating time and temperature. The active function of OVT, especially the iron-binding capacity, was highly dependent on its unique structure. OVT consisted of two similarly sized and homologous N and C leaflet structural domains, which can be further classified into two similarly sized sub-structural domains. These two sub-structural domains were linked by two antiparallel β-strands, allowing them to form either open or closed conformations. An iron-binding site was present in each sub-structural domain, so theoretically two iron ions could be bound per molecule of OVT [37]. Similarly to other transferrin proteins, the leaflet structural domains in the protein structure of OVT underwent significant conformational changes during iron ion binding and release. The cleft between the two substructural domains opened before binding iron ions and closed and became tight when iron ions were bound. The heating treatment may disrupt the iron-binding domains of some OVT molecules, leading to less flexibility in conformational changes, therefore reduced the iron-binding activity of OVT. Nevertheless, the results showed that OVT maintained most of its iron-binding capacity within a certain temperature range. pH was also a major factor affecting the binding of iron ions by OVT. Under acidic conditions, it was easily for the iron-binding OVT to release iron ions, whereas under alkaline conditions, the ability of OVT to bind iron ions was enhanced, which was in agreement with the results of the previous studies [38]. This could be explained by the fact that the structure of OVT changes under different pH conditions. In acidic environments, the protein portion of OVT was positively charged, which resulted in an electrostatic repulsion between it and positively charged iron ions, thus facilitating the release of iron ions. On the contrary, under alkaline conditions, the protein portion of OVT was negatively charged, which enhanced the electrostatic attraction between it and iron ions, thus improving the binding capacity of iron ions [39]. In addition, it has been reported that each iron ion could link to four residues on transferrin, two tyrosine (Tyr), one aspartic acid (Asp) and one histidine (His). Since Asp was an acidic amino acid, it carried a large negative charge when the ambient pH was alkaline. Tyr could also dissociate a phenolic hydroxyl group under alkaline conditions. The high net charge caused a strong intermolecular electrostatic repulsion, leading to the opening of the ring structure of OVT. Under these conditions, iron-binding sites were easily exposed, which facilitated the binding of positively charged Fe_3_^+^ to OVT. In addition, increasing the concentration of NaHCO_3_ in the solution also facilitated the increase in the iron-binding capacity of OVT. The interaction of OVT with bicarbonate was recognized as a prerequisite for iron uptake. This was similar to the findings of Chung et al. [40]. The first step was that amino acid residues around the ferric-binding site of the protein interacted with HCO_3_^−^. The second step was that environmental Fe^3+^ was attracted to the iron-binding site in the presence of the potentiating anion HCO_3_^−^.

### 3.10. Antioxidant Capacity of H-OVT

**Figure 7:**
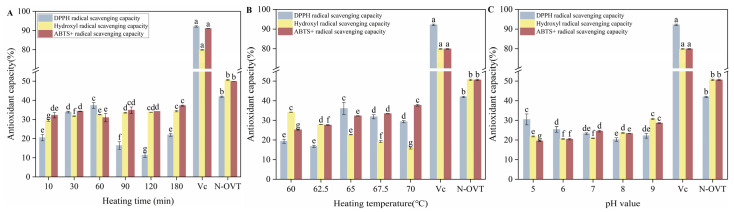
Antioxidant activity of OVT obtained by heat treatment at different times (**A**), temperature (**B**) and pH (**C**). (Different lowercase letters indicate statistically significant differences between groups).

Figure 7 illustrated the DPPH radical scavenging, hydroxyl radical scavenging and ABTS^+^ radical scavenging capacities of H-OVT obtained through heating at different times, temperature and pH. Vitamin C (Vc), also known as L-ascorbic acid, is a water-soluble vitamin. According to the results, Vc had the best antioxidant capacity. The antioxidant capacity of N-OVT was significantly lower than that of Vc. OVT treated with different temperatures and time showed significant differences in antioxidant activity. The DPPH radical scavenging capacity of H-OVT initially increased with prolonged heating time, peaking at 30 min (approximately 34%), followed by a decline (60–120 min) and subsequent recovery (120–180 min) (*p* < 0.05). The hydroxyl radical scavenging capacity of H-OVT gradually increased with elevated heating temperatures, but remained consistently lower than that of VC (approximately 85%) and higher than that of N-OVT (approximately 25%) across the tested range (*p* < 0.05). The ABTS^+^ radical scavenging activity exhibited a nonlinear variation over the tested time period, fluctuating around approximately 35% (*p* < 0.05) (Figure 7A). The DPPH scavenging capacity increased with a rising temperature, peaking at 65 °C, followed by a significant decline. Although lower than the positive control (VC, approximately 90%), heat-treated OVT (65 °C) exhibited significantly higher activity than untreated OVT (N-OVT, approximately 30%) (*p* < 0.05). The hydroxyl radical scavenging capacity gradually decreased with increasing temperature, indicating progressive structural alterations (*p* < 0.05). In contrast, the ABTS^+^ radical scavenging capacity increased progressively with elevated temperature, surpassing that of N-OVT (approximately 40%), but remaining substantially lower than VC (approximately 95%) (*p* < 0.05) (Figure 7B). The DPPH scavenging capacity increased with elevated temperature, reaching a peak at 65 °C before declining significantly. Although lower than the positive control (Vc, approximately 90%), heat-treated OVT (65 °C) demonstrated markedly higher activity compared to untreated OVT (N-OVT, approximately 30%) (*p* < 0.05). The hydroxyl radical scavenging capacity progressively decreased with increasing temperature (*p* < 0.05), suggesting gradual structural modifications. Conversely, the ABTS^+^ radical scavenging capacity rose steadily with temperature, exceeding that of N-OVT (approximately 40%) but remaining substantially lower than VC (approximately 95%) (*p* < 0.05) (Figure 7B). The reduced DPPH radical scavenging capacity of H-OVT may be attributed to protein hydrolysis via acidic or alkaline heat treatment, which alters or disrupts the amino acid sequence, size, and composition of peptides—factors known to influence DPPH radical scavenging activity [41]. The decrease in DPPH free radical scavenging activity also suggested that the electron-donating capacity of OVT was reduced after heat treatment. Similar results were reported for white bean protein [42]. On the other hand, after heat treatment, some antioxidant groups in OVT may be reduced or masked by heating, which may also be one of the reasons for the reduced scavenging activity of DPPH radicals [43]. In comparison, H-OVT showed higher overall activity in terms of hydroxyl radical scavenging ability and ABTS^+^ radical scavenging ability. You et al. found that duck egg ovalbumin had better ABTS^+^ radical scavenging ability but lower DPPH radical scavenging activity. The reason for this may be due to the fact that the samples were not high in hydrophobic amino acids, and thus reacted more readily in the water-soluble system, while reacting more slowly with the fat-soluble system DPPH [44]. The ABTS^+^ radical scavenging activity and hydroxyl radical scavenging activity of H-OVT were retained to a great extent, basically above 80%. Similar results were reported for heated pea isolate proteins, due to the fact that hydrogen and disulfide bonds may facilitate the reaction of proteins with free radicals [45]. Lee et al. found that the ABTS^+^ radical scavenging activity of OVT increased significantly in a dose-dependent manner after enzymatic hydrolysis [46]. In addition, the tripeptide IRW (Ile-Arg-Trp) purified from the OVT-hydrolyzed product showed better oxygen radical scavenging compared to the whole OVT-hydrolyzed product, which was attributed to the action of the peptide bond between Ile, Arg and Trp. The DPPH activity of H-OVT progressively decreased within the pH range of 5–9 (*p* < 0.05). In contrast, the hydroxyl radical scavenging capacity showed a slight linear increase from pH 5 to pH 9, with N-OVT (approximately 50%) being superior to pH-treated OVT. VC exhibited the highest activity (approximately 80%). The ABTS^+^ scavenging activity displayed an overall increasing trend across pH 5–9 (*p* < 0.05), with significant pH-dependent effects (Figure 7C). In an acidic environment, OVT displayed a relatively high scavenging capacity for DPPH free radicals. Meanwhile, hydrolyzed proteins possessed higher ABTS^+^ radical scavenging under alkaline conditions. This may be due to the fact that alkaline hydrolysis can effectively produce small active protein fragments [41].

## 4. Conclusions

Under specific temperatures, times and pH conditions, OVT can be extracted from egg white via moderate heat treatment. Single-factor experiments and response surface methodology were employed to validate and optimize the extraction parameters to achieve high-purity OVT with superior yield. Heat treatment under varying conditions altered OVT’s structure, physicochemical properties and bioactivity. Changes in solubility, particle size, zeta potential and PDI were primarily attributed to hydrophobic interactions (due to exposed hydrophobic regions) and electrostatic repulsion (from charge redistribution) following structural disruption. Secondary structure changes were driven by the breakage and formation of intramolecular hydrogen bonds and disulfide bonds during heat treatment, leading to altered proportions of α-helix, β-sheet and other structural elements. The bioactivity of OVT correlated with structural domain rearrangements and the partial hydrolysis of amino acids or peptides. Although heat-induced denaturation occurred, OVT retained key functionalities, including iron-binding and antioxidant activities. This study provides a novel method for efficient OVT extraction from egg white, laying the foundation for its industrial-scale production and offering insights into heat treatment effects on OVT’s physicochemical properties.

## Figures and Tables

**Table 1:** Test table of three factors and three levels.

Level	Factor		
	A Time (min)	B Temperature (°C)	C pH
1	30	60	6
2	60	62.5	7
3	90	65	8

**Table 2:** Orthogonal test results for extraction of OVT by heat treatment method.

Run				Factors			Yield (%)
	A	B	C	Time (min)	Temperature (°C)	pH	
1	−1	−1	0	30	60	8	85.64
2	1	−1	0	90	60	8	85.98
3	−1	1	0	30	65	8	83.72
4	1	1	0	90	65	8	85.50
5	−1	0	−1	30	62.5	7	82.16
6	1	0	−1	90	62.5	7	83.49
7	−1	0	1	30	62.5	9	83.61
8	1	0	1	90	62.5	9	84.38
9	0	−1	−1	60	60	7	88.33
10	0	1	−1	60	65	7	88.17
11	0	−1	1	60	60	9	88.80
12	0	1	1	60	65	9	89.81
13	0	0	0	60	62.5	8	92.10
14	0	0	0	60	62.5	8	92.27
15	0	0	0	60	62.5	8	92.66
16	0	0	0	60	62.5	8	92.34
17	0	0	0	60	62.5	8	91.82

**Table 3:** Response surface model analysis of variance of OVT yield.

Source	Sum of Squares	df	Mean Square	F	*p*	Significant
Model	211.12	9	23.46	95.54	<0.0001	Significant
A-A	2.23	1	2.23	9.07	0.0196	
B-B	0.3	1	0.3	1.24	0.3024	
C-C	2.46	1	2.46	10.04	0.0158	
AB	0.52	1	0.52	2.11	0.1895	
AC	0.078	1	0.078	0.32	0.5897	
BC	0.34	1	0.34	1.37	0.2801	
A2	161.20	1	161.20	656.56	<0.0001	
B2	2.85	1	2.85	11.60	0.0113	
C2	28.96	1	28.96	117.94	<0.0001	
Residual	1.72	7	0.25	4.41	0.0930	Not significant
Lack of Fit	1.32	3	0.44			
Pure Error	0.4	4	0.1			
Cor Total	212.83	16				
	R^2^ = 0.9904		R^2^adj = 0.9781		CV = 0.73	

A = time, B = temperature, C = pH.

## Data Availability

The original contributions presented in this study are included in the article. Further inquiries can be directed to the corresponding author.
